# Supplementary material for: Association between acculturation and physician trust for internal migrants: A cross-sectional study in China
Source: PLoS One. 2023 Mar 9;18(3):e0280767. doi: 10.1371/journal.pone.0280767 (PMC9997971; doi:10.1371/journal.pone.0280767)
Supplement: S1 Table — (DOCX) [file pone.0280767.s001.docx]

**S1 Table. Information about the legal permanent system or temporary residency permit system in China.**

| The legal permanent system or temporary residency permit system in China is truly quite complex.In general, Both systems work for two types of population:foreigners and Chinese citizens. The legal permanent system for the former is applicable for those who want a permanent residence ID card or a Chinese “Green Card”. With the card, an expatriate will be able to enter China just like Chinese citizens, without going through visa formalities.Also, some foreigners want temporary residence permits,which are required for them to stay for more than 6 months in China, but not more than a year. Various international visits are allowed with this type of residence permit. The legal permanent residency or temporary residency permit system is also applicable for Chinese citizens who migrate between regions of China,which is due to the hukou system, also known as the household registration system. It is a unique institutional feature of migration in China. It is loosely similar to an internal passport system that restricts rural migrants’ mobility and is linked to access to local welfare and resources (Zhang, 2010; Vendryes, 2011). There are two main types of hukou, an agricultural type and a non-agricultural type; this classification is based on the rural/urban classification of a person's birth place (Chan and Zhang, 1999).For internal migrants to urban cities, there are two options :holding the permanent residency permit(or local hukou of cities) or temporary residency permit (or juzhuzheng),and between them ,and a transformation from juzhuzhen to local hukou is introduced through points system. The residence permit points systems assesses the contribution of migrants to the city, their education and employment background, and property ownership. Those with the highest scores become eligible to apply for local hukou. Through the permits, and depending on their points, internal migrants have ‘equal rights’ to access state services including children’s education, employment services, health and family planning, culture and sports, legal aid, vehicle registration and vocational examinations. Taking Shanghai as a case, the internal migrants scoring above 120 points receive privileges, including in education — their children are allocated school places first after local hukou-holders, gaining entry to well-performing, centrally-located schools. Those with lower scores must wait for spaces in lower quality state schools and state-aided private schools on the city outskirts. Those without residence permits have no access to schooling in the city at all. |
| --- |
