# Supplementary material for: Association between acculturation and physician trust for internal migrants: A cross-sectional study in China
Source: PLoS One. 2023 Mar 9;18(3):e0280767. doi: 10.1371/journal.pone.0280767 (PMC9997971; doi:10.1371/journal.pone.0280767)
Supplement: S1 File — (DOCX) [file pone.0280767.s002.docx]

**The Survey Questionnaire**

**Individual characteristics**

1. Your age: ____ years height CM weight KG

2. your gender: Male Female

3. Your Marital Status: Unmarried , Married , Divorced , Widowed

4. Your education level: Lower or equal to primary education primary school ③ Secondary education ④College education ⑤ Higher or equal to graduated education

5. Your profession: Unemployed (unemployed at home) Government civil servant ③ White-collar worker ④ migrant worker ⑤ Self-employed ⑥ Retired ⑦ Others

6. Your insurance: No insurance New rural cooperative medical insurance Urban employee medical insurance Urban residents medical insurance ⑤Other insurance

7. Your place of origin: non-Shanghai Shanghai (if you choose ② , please answer directly from question 10)

8. When do you start to settle in this city? :  year month day

9. Have you obtained Shanghai’s hukou: YesNo

If "No" is selected, do you have a local residence permit (temporary residence permit): YesNo

10. Numbers of children in your family: 0, 1, 2 ④ 3 and more

11. Your family’s annual household income: below ￥50,000 ②￥50,000-100,000③￥100,000-250,000④ ￥250,000 and more

Do you have your own property: Yes No

13. The location you live in: the Metropolitan area Fringe areas ③Suburban areas

14. Annual physical examination frequency ① Never② Once ③ Twice ④ Three times and more

15. The number of times you have visited a doctor in past one year: 0 1 time 2 times ④ 3 times and more

**Acculturation**

1.What degree to which do you speak Shanghainese? ①Very well②Well ③ Not well④Not at all

2. What degree to which do you listen Shanghainese? Very wellWell ③ Not well④Not at all

3.The language you use at home: Shanghainese Mandarin ③Hometown dialect

4.The language you use at work: Shanghainese Mandarin ③Hometown dialect

5.The language you use when you are with your friends: Shanghainese Mandarin ③Hometown dialect

6.Changes in your diet after you came to this city:①Completely the same②Very similar③Somewhat different④Very different⑤Completely different

7.Changes in your dressing after you came to this city: ①Completely the same②Very similar③Somewhat different④Very different⑤Completely different

8.Changes in your entertainment after you came to this city: ①Completely the same②Very similar③Somewhat different④Very different⑤Completely different

9.Changes in your social customs after you came to this city: ①Completely the same②Very similar③Somewhat different④Very different⑤Completely different

**Doctor-patient relationship**

1. The following questions reflect your level of trust in physicians. Please indicate your degree of trust according to the following statements in the blanks. Please select only one item per box.

|  | Items | Strongly  agree | Agree | Disagree | Strongly  disagree | Don’t know |
| --- | --- | --- | --- | --- | --- | --- |
| 1 | I feel that the facilities, medical staff and service environment of the medical institution are good |  |  |  |  |  |
| 2 | I feel like the wait time for appointments and consultations is very long * |  |  |  |  |  |
| 3 | Whether your condition is mild or severe, the doctor can always pinpoint the problems |  |  |  |  |  |
| 4 | I am confident that the doctor's diagnosis is correct |  |  |  |  |  |
| 5 | Doctors think more about my health than treatment cost |  |  |  |  |  |
| 6 | My doctor puts my interests first, not his own or the hospital's |  |  |  |  |  |
| 7 | The dose and timing of the medicine given by the doctors are appropriate |  |  |  |  |  |
| 8 | Doctors sometimes do too many physical examinations * |  |  |  |  |  |
| 9 | Doctors care about patients and listen to patients |  |  |  |  |  |
| 10 | The doctor will give me the opportunity to ask him about his condition |  |  |  |  |  |
| 11 | I have confidence in the technical ability of the doctors in the hospital |  |  |  |  |  |
| 12 | I trust the hospital in general |  |  |  |  |  |

2. What do you think is the gap between the quality of health services actually received and the quality services:

(1) Very deep; (2) Deep; (3) General (4) Not deep; (5) The same

4. Based on your experience in seeing a doctor, what do you think the degreeof relationship between doctors and patients in Shanghai? (1) Very poor; (2)Relatively poor; (3) General (4) Relatively good (5)Very good

**Self-reported health status**

1.Accoding to your current actual health status, please make a self-assessment of your health status in the following options, and select one item from each row for "Evaluation before coming to Shanghai" and "Evaluation after coming to Shanghai".

| Classification | Items | Evaluation before coming to Shanghai | | | | | Evaluation after coming to Shanghai | | | | |
| --- | --- | --- | --- | --- | --- | --- | --- | --- | --- | --- | --- |
|  |  | very good | good | bad | very bad | Don’t know | very good | good | bad | very bad | Don’t know |
| physical health | Moderate intensity activity* |  |  |  |  |  |  |  |  |  |  |
| mental health | Emotional coordination |  |  |  |  |  |  |  |  |  |  |
|  | family coordination |  |  |  |  |  |  |  |  |  |  |
|  | friend coordination |  |  |  |  |  |  |  |  |  |  |
| social health | overall health |  |  |  |  |  |  |  |  |  |  |
|  | overall happiness |  |  |  |  |  |  |  |  |  |  |

Note: * Moderate-intensity activities such as moving tables, vacuuming or cleaning floors, bowling, or tai chi.

1. According to your current physical health condition, what are the numbers of the following disease types I: ①Asthma ②Back pain ③Hypertension ④Diabetes ⑤Allergies ⑥Migraines ⑦Ulcers ⑧bronchitis ⑨arthritis

(1) None of the above (2) 1 type (3) 2 types or more

1. According to your current physical condition, what are he numbers of the following disease types II: ① Heart disease ② Cancer ③ Thyroid disease
2. None of the above (2) 1 type (3) 2 types or more

This concludes the questionnaire, thank you for your participation. This questionnaire is an anonymous survey, and your content is strictly kept confidential.If you are interested in the results of this research, please leave your contact information here, and we will send you the final research findings and conclusions, which may be helpful to you. Thanks again for your participation

My contact details (optional):
