# Supplementary figures and images for: Association between acculturation and physician trust for internal migrants: A cross-sectional study in China
Source: PLoS One. 2023 Mar 9;18(3):e0280767. doi: 10.1371/journal.pone.0280767 (PMC9997971; doi:10.1371/journal.pone.0280767)

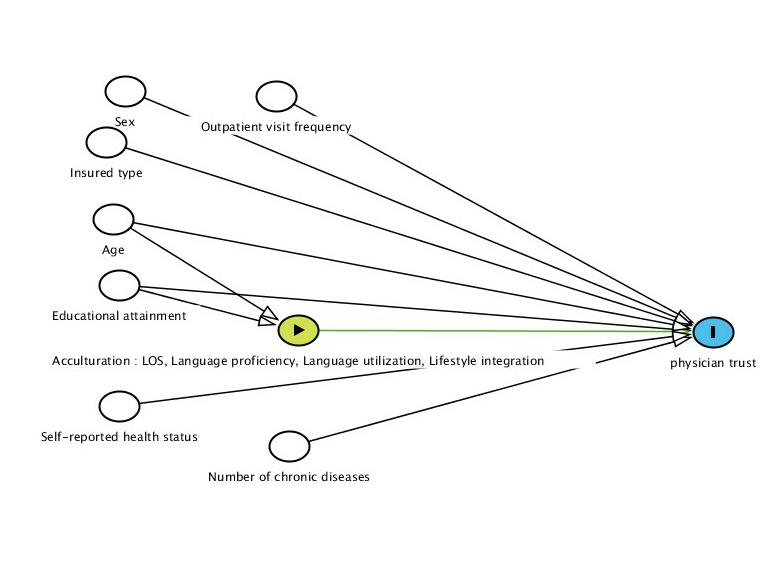

Supplement: S1 Fig — (JPG) [file pone.0280767.s003.jpg]
