# Supplementary material for: Association between acculturation and physician trust for internal migrants: A cross-sectional study in China
Source: PLoS One. 2023 Mar 9;18(3):e0280767. doi: 10.1371/journal.pone.0280767 (PMC9997971; doi:10.1371/journal.pone.0280767)
Supplement: Attachment — Submitted filename: Response to Reviewers.docx [file pone.0280767.s005.docx]

**A rebuttal letter**

Journal Requirements:

When submitting your revision, we need you to address these additional requirements.

1. Please ensure that your manuscript meets PLOS ONE's style requirements, including those for file naming. The PLOS ONE style templates can be found at

https://journals.plos.org/plosone/s/file?id=wjVg/PLOSOne_formatting_sample_main_body.pdf and <https://journals.plos.org/plosone/s/file?id=ba62/PLOSOne_formatting_sample_title_authors_affiliations.pdf>

**Response:we have revised the format of the manuscript according to the PLOS ONE's style requirements.**

2. PLOS ONE does not copy edit accepted manuscripts (https://journals.plos.org/plosone/s/criteria-for-publication#loc-5). To that effect, please ensure that your submission is free of typos and grammatical errors.

**Response:we have corrected typos and grammatical errors occurred in the manuscript through a writing screening of manuscript before re-submission.**

3. Thank you for stating the following in the Acknowledgments Section of your manuscript:

“Funding was provided by National social Science Foundation of China General Project (Grant No. 19BGL246); National social Science Foundation of China Major Project (Grant No. 18ZDA088); Shanghai 2021 "Science and Technology Innovation Action Plan" Soft Science Key Project (Grant No. 21692104900). The funders had no role in the question design, analysis or interpretation.”

We note that you have provided additional information within the Acknowledgements Section that is not currently declared in your Funding Statement. Please note that funding information should not appear in the Acknowledgments section or other areas of your manuscript. We will only publish funding information present in the Funding Statement section of the online submission form.

Please remove any funding-related text from the manuscript and let us know how you would like to update your Funding Statement. Currently, your Funding Statement reads as follows:

 “--E.D.  T.W.

--Grant No. 19BGL246 and Grant No. 21692104900 :E.D.;Grant No. 18ZDA088:T.W.

--National social Science Foundation of China General Project (Grant No. 19BGL246); National social Science Foundation of China Major Project (Grant No. 18ZDA088); Shanghai 2021 "Science and Technology Innovation Action Plan" Soft Science Key Project (Grant No. 21692104900)

--http://www.nopss.gov.cn/GB/index.html

http://stcsm.sh.gov.cn/

--The funders had no role in study design, data collection and analysis, decision to publish, or preparation of the manuscript.”

Please include your amended statements within your cover letter; we will change the online submission form on your behalf.

**Response: We have added the** Acknowledgments **and deleted funding statements int the manuscript.**

4. Your ethics statement should only appear in the Methods section of your manuscript. If your ethics statement is written in any section besides the Methods, please delete it from any other section.

**Response : we have revised it.**

[Note: HTML markup is below. Please do not edit.]

Reviewers' comments:

Reviewer #1: I think this is a policy-relevant research article. I thought that the definition of internal migrants may varied across time. The authors may give a clear objective study population under the circumstance of a cross-section data time.

**Response:Thank you for this suggestion. Accordingly,we have added the definiton of study population:internal migrants into the section of introduction. please see highlighted lines in that section.**

Reviewer #2: Thank you for considering me as a reviewer for this manuscript in your esteemed journal. In general, I believe that it’s an interesting study. The authors focus on an important research question. It also has a great potential for developing a practical, culturally safe tool that can be used to improve the patient experience of migrant workers and promote social cohesion in China. This study may also have implications for other countries that are experiencing the rise of urbanization. However, there are some key issues that I suppose the authors need to address before the manuscript can be considered for publication:

Major concerns:

Introduction

Motivation

1. I suggest our authors re-emphasize the importance of physician trust in health service research as well as its relationship with other important psychosocial measures that they have mentioned (e.g., physician-patient relationships, ratings of providers). For example, is there any evidence to show that physician trust has an impact on the health service utilisation or the health outcomes of patients? Is there any overlap between physician-patient relationships and physician trust?

**Response :According to your suggestion, we have supplemented the statements on the association of physician trust with healthcare utilization, health outcomes of patients and physician-patient relationship. Please see highlighted lines in the section of introduction.**

2. The authors mentioned that more than 60% of health care workplace violence in Shanghai was caused by migrant workers. It would have made the authors' research questions more meaningful if they had given the percentage of migrant workers in the population of Shanghai, or the percentage of migrant workers in outpatient/inpatient care utilisations.

**Response：Thank for your recommendations, we have added the percentage of the internal migrants on outpatient and emergency department visits to demonstrate the research questions pointed out by the reviewer. Please see details highlighted in the section of introduction.**

Conceptual framework

3. The authors should explain why “immigration acculturation theory” is suitable for studying migrant workers in China (since it was designed for immigrants in the Western world) and how they developed their framework (based on existing literature? Expert opinions? Or something else?). From my point of view, many structural barriers (e.g., hukou, health insurance, perceived discrimination) faced by migrant workers are not mentioned in this framework and it may have a nonnegligible impact on their cultural acculturation and trust in physicians. In addition, a clear definition of acculturation is needed. Based on the results, it seems that adjusted for demographic covariates, socioeconomic covariates, and health profiles, only the LOS in Shanghai, good speaking skills of Shanghai dialect, and lifestyle integration are significantly associated with trust. I’m not sure if LOS in Shanghai and the good speaking skills of Shanghai dialect are good indicators for acculturation. Maybe a long time stay and learning local dialect do make people get more familiar with the healthcare system in Shanghai and have more experiences navigating the system, but these performances do not necessarily mean that he/she recognizes or integrates into the local culture.

**Response: Thanks for your detailed suggestions. Based on the prior literature (listed below as 1-3), immigration acculturation theory is also suitable for studying migrant workers or internal migrants in China due to the reason that internal migrants, as a part of large indigenous population, were observed to encounter difficulties of adapting into the cities caused by the existing intra-cultural differences between different regions and some structural barriers when they migrated from rural to urban areas. We added this description in the section of introduction (highlighted part). Meanwhile, we added the definition of acculturation according to your suggestion. Moreover, some previous studies have applied the indicators of acculturation with LOS dialect proficiency, dialect utilization and lifestyle adaption. Please see the highlighted lines in the sub-section of Measures.**

[1]Miao S, Xiao Y. Does acculturation really matter for internal migrants’ health? Evidence from eight cities in China. Social Science & Medicine. 2020 Sep 1;260:113210.

[2]Fang L, Sun RC, Yuen M. Acculturation, economic stress, social relationships and school satisfaction among migrant children in urban China. Journal of Happiness Studies. 2016 Apr;17(2):507-31.

[3]Jihong Y.The Cultural Adaptation of New Urban Immigrants: A Case Study of Landless Farmers.Tianjin Social Sciences.2010;2:62-5.(Chinese)

4. The authors should also be aware of their ideological assumptions in the framework. For example, some research that used a critical theory framework may focus on structural inequality while others may concentrate on micro-level factors such as health literacy and focuses on psychosocial factors and cultural contexts. However, research focused on either side should focus on the concerns of the other side. For example, if we ignore the “oppressive structures” that migrant workers are facing, we may “lead to the pathologization of immigrants who may not be seen as acculturating ‘enough’, even if their behaviour is related to their experience of social exclusion or discrimination within the larger society”. (See Sakamoto, Izumi. "A critical examination of immigrant acculturation: Toward an anti-oppressive social work model with immigrant adults in a pluralistic society." British Journal of Social Work 37.3 (2007): 515-535.) Maybe the author could consider more variables about structural barriers to address this issue.

**Response: Thank for your suggestion. In this study, we applied the acculturation theory and examined the association of acculturation with physician trust among internal migrants in China under the assumption that these structural barriers did not present effects on the trust in physicians. We agreed with the reviewer that the acculturation theory has some deficiencies, which largely ignore structural issues affecting individuals’ ability in dealing with unfamiliar culture(s). As the reviewer mentioned, the theory does not capture the big picture behind acculturating individuals and may lead to pathologization of immigrants who may not be seen as acculturating ‘enough’. Therefore, variables on structural barriers such as cultural distance, perceived discrimination, and hukou should be carefully considered to address the issues. But due to the survey methods we employed, it is hard for us to add these variables at this time point. We will certainly take into consideration of these important variables in our future research. We added the reviewer’s suggestion as a part of the research limitations into the revised manuscript. Please see the statements highlighted in the section of discussion.**

Method

Data collection

1. The legal permanent system or temporary residency permit system in China is quite complex. Maybe the author could add more description in the appendix for readers to understand. It would also provide a better rationale for the sampling strategy.

**Response:We have added a supporting information file to describe the legal permanent system or temporary residency permit system in China. Please see details in the S1 Table.**

Measurement

2. The information about the questionnaire is inadequate, and the measurements of many variables are unclear. It would be useful to attach the questionnaire they used in the survey. For example, what questions the authors have asked for measuring “life integration”? And how this variable was dichotomized int to a binary variable (high vs low)?

**Response: According to your recommendations, we displayed the survey questionnaire in the supporting information file(S2 File). The measurements of many variables can be seen in this file. In addition, we added the measurement approach of the variables of interest in the study. Please see highlighted lines in the sub-section of measures of the revised manuscript.**

Statistical analysis

3. Please state the rationale for the covariates that you have controlled. A DAG may be helpful (http://www.dagitty.net/dags.html).

**Response: According to your recommendation,we have supplemented the rationale for the covariates in the study with a DAG figure,please see details highlighted in the sub-section of statistical analysis and Fig 2.**

Discussion & Conclusion

1. Hedonic recall bias: I believe the hedonic recall bias is more related to objective measurements, such as income. I wonder if the authors could show some evidence about the hedonic recall bias of psychosocial measurements.

**Response: Thank for your correction. We have revised it into “recall bias”, not “hedonic recall bias”, which more often occurs in the self-reported objective measurements.**

2. The authors argued that their results “represent all of the top 4 largest cities of China (Beijing, Shanghai, Guangzhou, and Shenzhen)”. I can’t agree since these four tier-1 cities in China are very different in terms of geography, economics, and culture.

**Response: We have explained this in the section of Contributions and policy implications. Please see the details highlighted in that position.**

Minor concerns:

1. To my knowledge, the word “indigenous population” is used to differentiate the Indigenous peoples of the Americas from the European settlers. I guess here the authors are referring to the “rural population”. Also, the word “migrant worker” may be a better choice for “internal immigrant”.

**Response: In this study, the internal migrants refer to a population that has been living in a place other than their officially registered residence (hukou) for at least 6 months. This population does not only include the migrant workers flowing from rural areas to urban cities, but also the population who flow from one urban city into another different urban city. Therefore, we identify internal migrant as the study population instead of migrant worker you recommended.**

2. The English language of the manuscript is not suitable for publication. For example, “the outstanding gap between the restricted supply of health service provided by the government and emergent medical demand from migrants” → “the large/huge gap between …” (Introduction).

**Response: As you suggested, we have fully revised the language of this manuscript by a local English-speaking peer in the current version.**

3. Typos: “(p-value < 0.5)” → “(p-value < 0.05)” (Method). “10,000-250,000” → “100,000-250,000”. The mean (or median?) age for the “Good physician trust” and “Not trust physicians” groups are missing. The “Acculturation” is categorized as “low/high”, but the figures have five rows (Table 1).

**Response: Thanks for your correction, we have revised these accordingly. Please see highlighted lines where these typos occurred.**

4. “Gender” is referring to men and women. “Sex” is usually categorized as female or male.

**Response: We have revised gender into sex.**

5. Since this study is focusing on the association between acculturation and physician trust, the authors should avoid using words that suggest causality such as “effect”, “cause”, “affect”, and “impact”.

**Response: as you recommended, we replace the word “effect” with “assocation” , and revised the title of the manuscript accordingly.**

6. Several other variables may play important roles in this study such as the birthplace and the hukou of participants. I am not sure whether the authors have these measurements, but it would be interesting to see if the place of birth or hukou status is confounding factors or effect modifiers.

**Response: We agreed that the place of origin and hukou for immigrants are important variables in the immigration studies. However, in this study, we extracted 1330 participants as internal migrants who have been living in Shanghai instead of their officially registered residence (hukou) for at least 6 months, which means that their birthplace was not Shanghai and they did not have Shanghai’s hukou, so we didn’t include the two variables in the study. This criterion of eligible participant inclusion was mentioned in the sub-section of participants of Materials and Methods.**
