# Supplementary material for: Association between acculturation and physician trust for internal migrants: A cross-sectional study in China
Source: PLoS One. 2023 Mar 9;18(3):e0280767. doi: 10.1371/journal.pone.0280767 (PMC9997971; doi:10.1371/journal.pone.0280767)
Supplement: Attachment — Submitted filename: Response to Reviewer.docx [file pone.0280767.s006.docx]

**Response to Reviewers**

6. Review Comments to the Author

Please use the space provided to explain your answers to the questions above. You may also include additional comments for the author, including concerns about dual publication, research ethics, or publication ethics. (Please upload your review as an attachment if it exceeds 20,000 characters)

Reviewer #2: I'm happy to see the authors have addressed most of my comments and significantly imporved their manuscript. Here are my comments for your to consider:

1. In DAGs, time flows left to right. We usually draw exposures on the left and draw outcomes on the right. For example, if you think the number of chronic diseases of patients changed their trust to physicians, you should put it on the left side of physician trust. Since DAG is not the main methodology or result in your paper, you can put it in the appendices.

**Response：Thank for your suggestion,we have adjusted the position of this variable, and put it in the appendix file S3.**

2. The authors still use the word "indigenous population" in the second paragraph of background. As I said in the previous round of review, the term indigenous first came into use by Europeans to describe Indigenous peoples of the Americas and this word is not suitable for internal migrants in China.
**Response:We have revised this statement for the word.**

3. This manuscript still needs extensive revision for language and grammar. For example: "Association of acculturation with physician trust" → "Association between acculturation and physician trust", "salient association" → "significant association", "cut-point" → "cut-off point(or cut-off value)", "Physician Trust Good" → "High level of Physician Trust", "first-tier cities" → "Tier-1 cities". In addition, do not use the word like "liberal p value".

**Response:We have a professional language editor review the manuscript before re-submission accordingly.**
